# A single high dose of dexamethasone affects the phosphorylation state of glutamate AMPA receptors in the human limbic system

**DOI:** 10.1038/tp.2016.251

**Published:** 2016-12-13

**Authors:** M W Lopes, R B Leal, R Guarnieri, M L Schwarzbold, A Hoeller, A P Diaz, G L Boos, K Lin, M N Linhares, J C Nunes, J Quevedo, Z A Bortolotto, H J Markowitsch, S L Lightman, R Walz

**Affiliations:** 1 Department of Biochemistry, Federal University of Santa Catarina, Floranópolis, Brazil; 2 Center for Applied Neuroscience, Hospital Universitário, Federal University of Santa Catarina, Florianópolis, Brazil; 3 Epilepsy Center of Santa Catarina, Federal University of Santa Catarina, Florianópolis, Brazil; 4 Neurosurgery Unit, Governador Celso Ramos Hospital, Florianópolis, Brazil; 5 Department of Internal Medicine, Federal University of Santa Catarina, Florianópolis, Brazil; 6 Anesthesiology Division, Hospital Governador Celso Ramos, Florianópolis, Brazil; 7 Department of Surgery, HU, Federal University of Santa Catarina, Florianópolis, Brazil; 8 Pathology Division, HU, Federal University of Santa Catarina, Florianópolis, Brazil; 9 Translational Psychiatry Program, Department of Psychiatry and Behavioral Sciences, McGovern Medical School, The University of Texas Health Science Center at Houston, Houston, TX, USA; 10 Center of Excellence on Mood Disorders, Department of Psychiatry and Behavioral Sciences, McGovern Medical School, The University of Texas Health Science Center at Houston, Houston, TX, USA; 11 Neuroscience Graduate Program, The University of Texas Graduate School of Biomedical Sciences at Houston, Houston, TX, USA; 12 Laboratory of Neurosciences, Graduate Program in Health Sciences, University of Southern Santa Catarina, Criciúma, Brazil; 13 Centre for Synaptic Plasticity, School of Physiology, Pharmacology and Neuroscience, University of Bristol, Bristol, UK; 14 Physiological Psychology, University of Bielefeld, Bielefeld, Germany; 15 Henry Wellcome Laboratories for Integrative Neuroscience and Endocrinology, School of Clinical Sciences, Faculty of Medicine and Dentistry, University of Bristol, Bristol, UK

## Abstract

Glucocorticoids (GC) released during stress response exert feedforward effects in the whole brain, but particularly in the limbic circuits that modulates cognition, emotion and behavior. GC are the most commonly prescribed anti-inflammatory and immunosuppressant medication worldwide and pharmacological GC treatment has been paralleled by the high incidence of acute and chronic neuropsychiatric side effects, which reinforces the brain sensitivity for GC. Synapses can be bi-directionally modifiable via potentiation (long-term potentiation, LTP) or depotentiation (long-term depression, LTD) of synaptic transmission efficacy, and the phosphorylation state of Ser831 and Ser845 sites, in the GluA1 subunit of the glutamate AMPA receptors, are a critical event for these synaptic neuroplasticity events. Through a quasi-randomized controlled study, we show that a single high dexamethasone dose significantly reduces in a dose-dependent manner the levels of GluA1-Ser831 phosphorylation in the amygdala resected during surgery for temporal lobe epilepsy. This is the first report demonstrating GC effects on key markers of synaptic neuroplasticity in the human limbic system. The results contribute to understanding how GC affects the human brain under physiologic and pharmacologic conditions.

## Introduction

Stress responses initiated by environmental threats promote autonomic, endocrine, and behavioral changes that help self-preservation.^1^ The prefrontal cortex (PFC), amygdala (AMY) and hippocampus (HIP) are the key brain structures of the feedforward and feedback networks that mediate states of stress and fear. Glucocorticoids (GC) released during stress responses have feedforward effects in the whole brain, with a particular importance in limbic structures.^1, 2^ During non-stress conditions, the PFC exerts top–down regulation of limbic structures including the AMY, but in acute stress bottom–up processes prevail and behavior changes from slower, highly flexible responses to faster, stereotyped reaction.^3^ Stress can be helpful or harmful depending on its intensity, duration and personal features.^2, 4^ In predisposed individuals acute and intense stress has been associated with post-traumatic stress disorder,^2, 4^ and chronic and repetitive stress with other psychiatric disorders, including anxiety and mood disorders.^2, 5^ In rodents the stress and excessive GC release reduce synaptic plasticity and dendritic spines in the PFC and HIP, impairs hippocampal neurogenesis, and increase synaptic plasticity and dendritic alterations in the AMY, which has been associated with behavioral abnormalities.^5, 6, 7^ In humans, evidence correlates the morphological, metabolic, functional, and cognitive effects of GC on limbic structures.^8, 9, 10, 11, 12^ The brain sensitivity to GC, particularly the limbic system, may contribute to the high incidence of GC-related neuropsychiatric side effects.^13^ Furthermore, increased endogenous GC levels in Cushing's syndrome are commonly associated with depression, mania and anxiety.^14, 15^

Dependent on the applied stimulation frequency, active synapses are bi-directionally modifiable in mammalian brain regions such as the AMY, HIP and neocortex.^16, 17, 18^ The long-lasting increase of synaptic transmission, called long-term potentiation (LTP), is induced by high-frequency neuronal stimulation.^16, 18, 19, 20, 21^ Decreases in synaptic efficacy are also needed to reset the synapses, and are accounted by long-term depression (LTD), after low-frequency stimulation.^16, 17, 18^ Pharmacological evidence ‘*in vivo*' suggests an association between LTP and one-trial inhibitory avoidance,^22, 23, 24^ a fear associative memory task that induces LTP in the HIP.^25^ The fear conditioning, another fear associative memory task, can be inactivated and reactivated by LTD and LTP in the AMY, supporting a causal link between these synaptic processes and memory.^18^ Memory consolidation for both these tasks also can be modulated by GC.^1, 6, 26, 27, 28^

AMPA (α-amino-3-hydroxy-5-methyl-4-isoxazolepropionic acid) receptors (AMPARs) are heterotetrameric assemblies of GluA1–GluA4 subunits, usually permeable to Na^+^ and K^+^. Expression of Ca^2+^-permeable AMPARs, lacking GluA2 subunits (that is, GluA1 homomers) exist especially in the extrasynaptic and intracellular locations but can be recruited to synapses during neuroplasticity.^29^ Phosphorylation and dephosphorylation states of distinct sites of the GluA1 subunit of the AMPAR regulates the channel conductance and GluA1 synaptic membrane insertion is involved in the LTP and LTD induction.^29, 30, 31, 32, 33, 34^ Two major sites of GluA1 phosphorylation of AMPAR are studied regarding their role in neuroplasticity: (1) the Ser831, phosphorylated by Ca^2+^/calmodulin-dependent protein kinase II (CaMKII) and protein kinase C (PKC), which increases the channel conductance; (2) the Ser845, phosphorylated by cAMP-dependent protein kinase (PKA) that affects the open-channel probability of the receptor and regulates the synaptic incorporation of GluA1 subunit of AMPARs. It was reported that in naive synapses, LTD induction dephosphorylates Ser845, although in potentiated synapses the Ser831 is dephosphorylated by LTD induction. Conversely, LTP induction in naive synapses increases phosphorylation of GluA1-Ser831, whereas in depressed synapses phosphorylation of GluA1-Ser845 was enhanced.^34^ Serine 845 and 831 are dephosphorylated by low-frequency stimulation (LTD) in a manner dependent of protein phosphatase activity including PP1, PP2A and PP2B.^32, 35, 36^ Mice generated with knockin mutations in the GluA1 phosphorylation sites that shows deficits in LTD and LTP in the CA1 region of Hippocampus and have memory defects in spatial learning tasks. These results demonstrate that phosphorylation of GluA1 is critical for LTD and LTP expression and is involved in memory processing.^37^ Therefore, GluA1 phosphorylation of AMPARs sites at Ser831 and Ser845 sites are critical for ‘*in vivo*' synaptic plasticity being an emerging focus as a major target for stress and GC in the limbic system.^38, 39, 40^

The GC effects on the phosphorylation state of GluA1-AMPA, has never been investigated in the human limbic system. Mesial temporal lobe epilepsy related to hippocampal sclerosis (MTLE-HS) is the most common surgically remediable epileptic syndrome.^41^ Because surgery involves a ‘standard' resection of the neocortical and mesial temporal lobe structures,^41^ it offers an ethical opportunity to obtain human samples of AMY and HIP under well-controlled conditions to investigate phosphorylation state of proteins. Here we present a quasi-randomized controlled study showing the effect of a single high dose of dexamethasone (DEXA), on the phosphorylation levels of GluA1-Ser845 and GluA1-Ser831 in the human AMY, head of HIP and middle temporal neocortex gyrus (CX) of surgical patients with drug resistant MTLE-HS.

## Materials and methods

### Patients

We included 31 adult drug-resistant MTLE-HS patients treated surgically between May 2009 and December 2012 at the Centro de Epilepsia de Santa Catarina. They participated in a prospective study about synaptic plasticity markers in MTLE-HS. All patients had seizures that impaired awareness at least once a month (mean 7.5 per month), despite adequate treatment with at least two antiepileptic drugs (AEDs) in monotherapy. Their medical history, seizure semiology, neurological examination, psychiatric and neuropsychological evaluation, interictal and ictal surface video-EEG, and magnetic resonance imaging findings were consistent with unilateral MTLE-HS.^41, 42, 43, 44, 45, 46, 47^

Controlled clinical variables included gender, race, side of HS, age, disease duration, age of recurrent seizures onset, psychiatric comorbidities and quality of life.^41, 42, 43, 44, 45, 46, 47^ The AEDs used were carbamazepine, phenobarbital, diphenilhydantoin, valproic acid, lamotrigine or topiramate, associated or not with benzodiazepines (clobazam or clonazepam). The protocol was approved by the Ethics Committee for Human Research of Universidade Federal de Santa Catarina (365-FR304969). Written informed consent was obtained from all patients.

### Anesthesia protocol

The anesthetic protocol, except the DEXA treatment, were the same for all patients. Anesthesia started between 0730 hours to 0830 hours with intravenous (i.v.) bolus of propofol (2 mg kg^−1^), fentanyl (2 μg kg^−1^) and rocuronium (0.9 mg kg^−1^), followed by i.v. remiphentanil infusion (0.1 to 0.2 μg kg^−1^ min) and isofluorane inhalation (0.5 to 0.6 M.A.C.). Hydration was done with isotonic saline (1.2 ml kg^−1^ h) plus the half volume of diuresis. Cephalotine (30 mg kg^−1^) was given 30 min before the anesthesia. Oral AEDs were maintained until the day of surgery (0600 hours). Patients received 20 mg kg^−1^ of phenytoin i.v. 12 h before the surgery and 5 mg kg^−1^ i.v. after the brain samples were collected. Patients under phenytoin at home received only their morning oral dose and the intraoperative dose after the brain samples were collected.

### Dexamethasone treatment and study design

After the first 11 patients were included in the study, the anesthesiology team decided to use DEXA (10 mg i.v. bolus) immediately after intubation as an adjunctive anti-inflammatory and anti-emetic therapy.^48^ The DEXA infusion was not based on any clinical data. This change in the anesthesia protocol gave us the opportunity to design this quasi-randomized controlled study. The DEXA dose was calculated dividing 10 mg by the patient weight. The mean (s.e.) DEXA dose was 0.1575 (0.006)  mg kg^−1^ (range 0.11 to 0.2). Although this selective GR agonist differs from endogenous cortisol in many aspects of its transcriptional activity, this dose results in at least 27 times the effect of a daily adult human secretion of cortisol.^49^

### Surgery, intraoperative variables and brain tissue sampling

The surgeries and tissue sampling were done by the same neurosurgeon (MNL) and the principal investigator (RW) as previously described.^50^ The samples came from the brain tissue removed during a standard anterior and temporal lobectomy (ATL) procedure and were immediately frozen in liquid nitrogen and stored in −80 ^o^C freezer until the analyses. The sampling course is presented in Supplementary Figure 1. The temporal lobe resection included the middle and inferior temporal gyri extended up to 4 cm posterior from the temporal pole. Prior to the cortical resection, a 1 cm^2^ sample of the CX localized 3 cm posterior to the temporal pole was gently dissected from the white matter. After assessing the mesial temporal region, 2/3 of the AMY were collected including its basal and the lateral nucleus. Both AMY and CX were collected without previous thermocoagulation. After AMY resection, the HIP was removed ‘*en bloc*' and its head and part of the body were quickly dissected on ice-refrigerated glass. The time of HIP manipulation since the electrocoagulation of its vascular supply start until its resection was controlled. Arterial blood gases, electrolytes, hematocrit, hemoglobin, acid-basic, mean arterial pressure heart and respiratory rate parameters during the AMY/HIP sampling were controlled. The anesthesia duration until each brain sample was controlled as well. The hemodynamic and respiratory parameters remained stable and no surgical complication was reported.

### Biochemical analysis

Biochemical analysis was blinded for all clinical data. All samples were homogenized by the same researchers (MWL) in the same day, placed in liquid nitrogen, and storage at −80 ^o^C until the analysis. The quantification of phosphorylation levels and total amount of the target proteins were performed by western blot as we described previously.^51, 52, 53, 54, 55, 56^ Protein content was determined by Peterson's method.^57^ Proteins were detected after overnight incubation with specific antibodies diluted in TBS with tween with 2% bovine serum albumin in a 1:1000 dilution (anti-phospho-GluA1-Ser831 (Sigma-Aldrich, St Louis, MO, USA, A4352); anti-phospho-GluA1-Ser845 (Sigma-Aldrich, A4477); anti-total-GluA1 (Santa Cruz Biotechnology, Santa Cruz, CA, USA, sc-13152); anti-EAAT1 (Cell Signaling, Beverly, MA, USA, #5684); anti-EAAT2 (Cell Signaling,#3838); anti-PP1ca (Santa Cruz Biotechnology,sc-7482); anti-phospho-CaMKII (Cell Signaling, #3361); anti-total-CaMKII (Cell Signaling, #3362); anti-GFAP (Cell Signaling, #3670) and anti-total-AKT (Cell Signaling, #9272)); 1:2000 (anti-phospho-ERK1/2 (Sigma-Aldrich, M8159); anti-phospho-AKT (Sigma-Aldrich, P4112); anti-phospho-PKA substrates (Cell Signaling, #9624) and anti-phospho-PKC substrates (Cell Signaling, #2261)), 1:5000 (anti-phospho-JNK p54/46 (Sigma-Aldrich, J4750) and anti-total-JNK p54/46 (Sigma-Aldrich, J4500)); 1:10 000 (anti-phospho-p38MAPK (Merck-Millipore, Temecula, CA, USA, 05-1059) and anti-total-p38MAPK (Sigma-Aldrich, M0800)) and 1:40 000 (anti-total-ERK1/2 (Sigma-Aldrich, M5670)). All membranes were incubated with anti-β-actin (Santa Cruz Biotechnology, sc-47778, 1:2000) antibody to control the protein load for each sample on the gel. The phosphorylation level was determined as a ratio of the optic density (OD) of the phosphorylated band/the OD of the total band. The immunocontent was determined as a ratio of the OD of the protein band by the OD of the β-actin band. The bands were quantified using the Scion Image software (Frederick, MD, USA).

Owing to the lack of adequate brain tissue samples from healthy controls and to avoid intra-day biases of western blot quantification, an internal control (IC) coming from three pooled HIP homogenized in the same way and time as all the studied samples was applied in all electrophoresis. The strategy also allows comparisons of variations in the phosphoprotein percentage according to brain structures and clinical parameters among the patients. The OD ratio (phospho/total or total/β-actin) for each target protein in the IC was considered 100%. The OD ratio of each protein analyzed in the samples was correlated as a percentage of the IC. When ERK1 phosphorylation was analyzed, for example, and the OD ratio (OD phospho-ERK1/OD total-ERK1) of the IC was 0.75 and the OD ratio for the investigated sample was 0.9; this means that the OD ratio of the investigated sample was 120% of the IC.

### Statistical analysis

All the neurochemical and clinical variables showed a parametric distribution (Kolmogorov–Smirnov). The distribution of neurochemical, clinical and laboratory variables according to DEXA treatment were analyzed by the Student's *t*-test, analysis of variance or Fisher's exact test. Comparisons of GluA1-Ser831 and GluA1-Ser845 phosphorylation levels in the CX, AMY and HIP between patients who received or not DEXA treatment were analyzed by Student's *t*-test. To reduce the possibility of a type I error due to multiple comparisons (two GluA1 phosphorylation sites in three structures) the level of significance should be *P*<0.0085. Multiple linear regressions analyses were done to determine the independent effect of DEXA on the variation of the phosphorylation levels of the serine 831 and 845 of the GluA1 subunit of glutamate AMPARs. Variables showing associations with *P*<0.15 in the univariate analysis were included in the multiple linear regressions analysis. For the final regression model the *P*<0.01 were considered statistically significant.

## Results

Table 1 shows the clinical, demographic, and laboratory variables of patients according to DEXA treatment. There were trends for imbalances between DEXA and non-DEXA groups according to gender (*P*=0.13), AEDs regimen (*P*=0.04), phenobarbital use (*P*=0.13), monthly frequency of seizures (*P*=0.04), intraoperative serum glucose (*P*=0.10) and storage time of samples (*P*=0.05).

The phosphorylation profile according to DEXA groups is shown in Table 2. The AMY levels of P-GluA1-Ser831 were 20.9% lower in the DEXA-treated patients in comparison with non-DEXA group (*P*=0.0003). There were trends for lower levels of P-GluA1-Ser831 in the CX (*P*=0.06) and HIP (*P*=0.10) among patients treated with DEXA. There was a trend higher P-GluA1-Ser845 levels in the CX (*P*=0.02), but not in the HIP and AMY of DEXA-treated patients (Table 3).

The Supplementary Figure 2a shows a dose-dependent effect of DEXA on P-GluA1-Ser831 levels in the AMY (*r*=0.69; *r*^2^=0.48; *P*=0.0002). Because the non-normal distribution of DEXA dose related to patients who did not receive DEXA, a linear regression also was performed only with patients who received DEXA (*n*=21; Supplementary Figure 2b). This analysis confirmed the dose-dependent decrease of P-GluA1-Ser831 levels in the AMY by DEXA treatment (*r*=0.66; *r*^2^=0.43; *P*=0.002). The observed association was not affected by the order of surgery (*P*=0.79), reducing the possibility of non-identified confounders related to tissue sampling of non-DEXA group (data not shown).

To exclude confounding biases resulting from imbalances in the distribution of gender, AEDs regimen, phenobarbital use, frequency of seizures, intraoperative serum glucose and storage time of samples, the association between the AMY levels of P-GluA1-Ser831 and these variables were analyzed together with DEXA treatment. There were trends for association between AMY levels of P-GluA1-Ser831 and gender (*P*=0.02) and serum glucose (*P*=0.12) (top of Table 3). After a multiple linear regression (bottom of Table 3), only DEXA treatment remained independently associated with P-GluA1-Ser831 levels in AMY. The trend for association between DEXA treatment and P-GluA1-Ser831 and P-GluA1-Ser845 levels in the CX and P-GluA1-Ser845 levels in the HIP became non-significant (*P*>0.15) after controlling for imbalances in the clinical variables distribution by multiple linear regressions (data not shown).

The rapid effect of DEXA on the AMY levels of P-GluA1-Ser831 could be non-genomic changes on the PKC activity and P-CaMKII levels, or imbalance of PP1 levels between DEXA and non-DEXA groups. Table 4 shows that DEXA treatment decreases the mean levels of P-CaMKII in the AMY (*P*=0.02) by 22.4%, despite a trend (*P*=0.08) for higher levels of the total CaMKII in the DEXA treated patients. There were no differences in the PP1ca levels or PKC activity in the AMY between DEXA and non-DEXA groups. DEXA also had no effect on the AMY levels of PKA activity, P-ERK1, P-ERK2, P-JNK1, P-JNK2, P-AKT and P-p38 (*P*>0.29), excluding a non-specific effect of DEXA on kinases in general.

MTLE-HS patients show a variable degree of AMY neuronal loss and gliosis.^54^ We controlled the AMY levels of total GluA1 subunits. Variations in the astrogliosis were controlled determining the levels of GFAP and astrocytes glutamate transporters (EAAT1/2). There were no differences in the AMY levels of GluA1 subunits, GFAP, EAAT1 and 2 between DEXA and non-DEXA groups (Table 4). Representative western blot results are shown in the Supplementary Figure 3.

## Discussion

To the best of our knowledge, this is the first report showing the GC effects on synaptic neuroplasticity biomarkers in the human limbic system. We show that 4 h after a high dose of DEXA the AMY levels of P-GluA1-Ser831, but not P-GluA1-Ser845, decrease significantly in MTLE-HS patients. These findings indicate that DEXA treatment shifts the serine 831 residue of the GluA1-AMPAR to a dephosphorylated state in the AMY. Notably, in the same structure we also observed a reduction in the levels of P-CaMKII(Thr286), indicating a reduction in the autonomous activity of CaMKII activity.^32, 58, 59^ DEXA did not affect the PKC activity or PP1ca levels in AMY.

The effects could not be attributed to imbalances in the distribution of demographic, clinical, radiological, intraoperative and neurochemical variables of our patients. A trend for lower levels of P-GluA1-Ser831 was also observed in the HIP and CX, but the possibility of a false negative result related to the small sample size cannot be completely ruled out.

The AMY is particularly sensitive to rapid responses to GC both in animals^60, 61^ and in man.^62^ Furthermore, in an *ex vivo* rodent study, using lower concentrations of exogenous GC than in the current study, we have shown that both a stressor or the administration of exogenous GC affects both the physiology of AMPARs and neuroplasticity.^40^ Brief restraint or DEXA administration to rats increases the surface expression of GluA1 (but not the GluA2 subunit) and the magnitude of electrically induced LTP in the HIP. Furthermore, 60 min after the restraint stress or slice incubation with corticosterone or DEXA for 30 min the serine 845 residue phosphorylation levels of the of GluA1 subunit increases in the HIP. The effect is dependent on GC receptors and PKA, but independent of NMDARs.^40^ Corticosterone increases GluA2-AMPAR surface mobility in a time-dependent manner (peak in 15 min), thereby conditioning the extent to which chemical LTP stimuli effectively increase GluA2 synaptic content during synaptic potentiation in the HIP.^39^ In addition, corticosterone also increases GluA1-AMPAR surface trafficking.^39^ The phosphorylation state of Ser831 and Ser845 of GluA1-AMPAR was variable according to different studies, depending on the brain region, stress type applied or duration and sampling time after stress.^63^

Karst *et al.*^64^ described that the exposure to two pulses of corticosterone (10 to 20 min pulse duration with 1 to 3 h pulse interval) enhances the miniature excitatory postsynaptic currents (mEPSC) frequency in CA1 pyramidal cells of mice. By contrast, basolateral amygdala (BLA) neurons responded to the first pulse with increased mEPSC frequency, but with a decreased mEPSC frequency to a second pulse. Furthermore, in BLA cells from mice exposed to restraint stress before slice preparation, corticosterone rapidly decreased mEPSC frequency, an effect that is dependent on the non-genomic activation of mineralocorticoid receptors. Although cross-species implications can be problematical, it is certainly worth pointing out that our subjects would undoubtedly have had some degree of pre-surgical hospitalization stress prior to receiving DEXA with subsequent reduction of the AMY levels of P-GluA1-Ser831. This is a neurochemical marker which has been associated with the synaptic depotentiation to the naive state from a potentiated state. Interestingly, compatible with a rapid non-transcriptional effect, Lovallo *et al.*^65^ showed a reduction of the BOLD signals in both the AMY and HIP 15–18 min after the injection of a small hydrocortisone dose. The relationship between this GC-related effect on BOLD signal and the synaptic potentiation or AMPA phosphorylation state in the limbic system is unknown. The AMY is a central structure in the processing of emotional components of memory, coding social and biological meanings of events.^66, 67, 68, 69, 70^ Clinical findings suggest that the AMY is necessary for modulating negative and positive arousing stimuli during encoding, indicating its involvement in processing biologically relevant stimuli independently of their valence.^67, 68^ These hypotheses received support by recent findings showing the BLA is a site of divergence for circuits mediating positive and negative emotional or motivational valence.^69^ The role of GC on the synaptic plasticity of neurons participating in the positive and negative valence-circuits deserve further investigation.

The results help not only to understand the mechanisms involved in the human brain modulation by the stress hormones, but also the common side effects related to a worldwide frequently prescribed class of pharmaceuticals. The DEXA dose used in our patients (or an equipotent dose of other GC) is commonly used in clinical practice. A large study examining the effects of oral GC treatment (*n*=786 868 courses) showed an overall incidence of 15.7 per 100 person-years at risk of adverse neuropsychiatric outcomes, and 22.2 per 100 person-years at risk for patients on their first course of GC^13^ The outcomes included depression, delirium, mania, panic disorder and suicidal behavior. Considering the critical role of the GluA1 phosphorylation at Ser831 and Ser845 sites for *in vivo* synaptic plasticity,^18, 25, 37^ we believe the observed effect of DEXA in the limbic system of our patients may be, at least in part, related to the high incidence of adverse neuropsychiatric side-effects during GC treatments. This hypothesis is in agreement with a previous study showing the association between the GRIA1 gene, that encode the GluR1 AMPAR, and psychiatric disorders. A combined linkage analysis of 60 families from National Institute of Mental Health Bipolar Genetics Initiative (NIMH-BPGI) suggested an association between a SNP in the second intron on the *GRIA1* gene and psychotic bipolar disorder.^71^ A case–control study showed that two specific polymorphisms for the GRA1 were associated with schizophrenia in Italians.^72^

Recently the role of the dually phosphorylated GluR1 AMPAR at S831 and S845 on synaptic plasticity was questioned by Hosokawa *et al.*^73^ Using Phos-tag SDS-PAGE they found the majority of synapses did not contain any phosphorylated AMPAR and the amount of phosphorylated GluA1 was very low. Although the neuronal stimulation (chemical LTP) and learning (inhibitory avoidance) increased phosphorylation, the proportion also was still low. In contrast, Diering *et al.*^74^ using a variety of measurement methods, showed a large fraction of synapses positive for phospho-GluA1–containing AMPARs, were highly responsive to numerous physiologically relevant ‘*in vivo*' and ‘*in vitro*' stimuli. Their results support the large body of research indicating a prominent role of GluA1 phosphorylation in synaptic plasticity. This controversy has no implications for the analysis of our results because we demonstrated significant changes in the percentage of the GluA1 subunit phosphorylation that was controlled for the total amount of phosphorylated AMPAR in both investigated groups.

There are, of course, limitations to our study. We cannot exclude the observed effects of DEXA could be, at least to some degree, related to the epilepsy background and the related pathologic, clinical and pharmacological variables not occurring in people without epilepsy. The possibility of false negatives resulting from the relatively small sample size and the limitation to one single time point for tissue sampling need to be considered. This limitation is inherent to western blot studies applied to proteins phosphorylation analysis in which all samples must be homogenized in the same day and conditions to avoid significant inter-day variability. The inclusion of different time points of DEXA treatment is not clinically feasible considering the patients receive the DEXA treatment at the anesthesia induction and the tissue sampling was dependent of the time of surgery itself. The results come from patients with an epileptic syndrome showing structural and functional neuroplasticity changes including a variable loss of neurons and gliosis in the analyzed structures.^42, 75^ The possible imbalances of structural changes between the investigated groups were controlled by corrections for the protein amount, distribution of gliosis markers, glutamate transporters and glutamate subunit receptors. The criticism about the non-use of a classic randomization is minimized because: (i) patients were included consecutively and the treatment allocation was not based on any predetermined variable or specific clinical indication and; (ii) data collection was done in a prospective way, under exhaustive cautions about the confounding variables following a predetermined approved research protocol.

To summarize, a single high dose of i.v. DEXA reduces significantly the levels of P-CaMKII and P-GluA1-Ser831 in the AMY of MTLE-HS patients in a dose-dependent manner. These effects on the signal transduction molecules and synaptic neuroplasticity in the limbic system contribute to a better understanding the GC effects in the human brain under physiologic and pharmacologic conditions.

## Figures and Tables

**Table 1:** Clinical, demographic, neuroradiological, neurophysiological and surgical variables of patients with mesial temporal lobe epilepsy related to hippocampal sclerosis according to dexamethasone treatment

*Variables*	*All cases*, n=*31*	*No dexamethasone*, n=*11*	*Dexamethasone*, n=*20*	P*-value*
*Gender*				
Female	18 (58.1)	4 (36.4)	14 (70.0)	
Male	13 (41.9)	7 (63.6)	6 (30.0)	**0.13**
				
*Race*				
Caucasian	27 (87.1)	9 (81.8)	18 (90.0)	
Others	04 (12.9)	2 (16.2)	2 (10.0)	0.60
				
*Marital status*				
Single	17 (54.8)	8 (72.7)	9 (45.0)	
Married	10 (32.3)	2 (18.2)	8 (40.0)	
Divorced or widower	4 (12.9)	1 (9.1)	3 (15.0)	0.33
				
*Current work activity*				
Working	11 (35.5)	4 (36.4)	7 (35.0)	
Health insurance	4 (12.9)	7 (63.6)	9 (45.0)	
Not working	16 (51.6)	0 (0.0)	4 (20.0)	0.26
				
*History of initial precipitant injury*				
No	7 (22.6)	3 (27.3)	4 (20.0)	
Yes	24 (77.4)	8 (72.7)	16 (80.0)	0.68
				
*Family history of epilepsy*				
No	12 (38.7)	4 (36.4)	8 (40.0)	
Second-degree or distant	10 (32.3)	2 (18.2)	8 (40.0)	
First- degree	6 (19.4)	3 (27.3)	3 (15.0)	
Unknown	3 (9.7)	2 (18.2)	1 (5.9)	0.41
				
*MRI hippocampal sclerosis*				
Right side	16 (51.6)	4 (36.4)	12 (60.0)	
Left side	15 (48.4)	7 (63.6)	8 (40.0)	0.27
				
*Antiepileptic drugs regimen*^a^				
Monotherapy	9 (29.0)	6 (54.5)	3 (15.0)	
Two or more drugs	23 (71.0)	5 (45.5)	17 (85.0)	**0.04**
				
*Benzodiazepines*				
Yes	15 (48.4)	7 (63.6)	9 (45.0)	
No	16 (51.6)	4 (36.4)	11 (55.0)	0.46
				
*Antiepileptic drugs*				
Carbamazepine				
No	6 (19.4)	4 (36.4)	2 (10.0)	
Yes	29 (80.6)	7 (63.3)	18 (90.0)	0.16
* *Phenobarbital				
No	19 (61.3)	9 (81.2)	10 (50.0)	
Yes	12 (38.7)	2 (18.2)	10 (50.0)	**0.13**
Diphenilhydantoin				
No	28 (90.3)	11 (100.0)	17 (85.0)	
Yes	3 (9.7)	0 (0.0)	03 (15.0)	0.54
Valproic acid				
No	29 (93.5)	9 (81.8)	18 (90.0)	
Yes	4 (12.9)	2 (18.2)	2 (10.0)	0.60
Lamotrigin				
No	27 (87.1)	9 (81.8)	18 (90.0)	
Yes	4 (12.9)	2 (18.2)	2 (10.0)	0.60
Topiramate				
No	29 (93.5)	11 (100.0)	18 (90.0)	
Yes	2 (6.5)	0 (0.0)	2 (10.0)	0.53
				
*Hand dominance*				
Right	27 (87.1)	9 (81.8)	18 (90.0)	
Non-right	4 (12.9)	2 (18.2)	2 (10.0)	0.60
				
*Psychiatric comorbidities*				
No diagnosis	14 (45.2)	4 (36.3)	10 (50.0)	
Depressive disorder	8 (25.8)	2 (18.2)	6 (30.0)	
Anxiety disorder^b^	3 (9.7)	2 (18.2)	1 (5.0)	
Other psychiatric conditions^c^	6 (19.4)	3 (27.3)	3 (15.0)	0.48
				
*Mean (s.e.)*				
Age (years)	36.4 (2.2)	34.6 (3.8)	37.3 (2.7)	0.57
Education (years)	6.6 (0.5)	6 (1.0)	6.9 (0.6)	0.43
Disease duration (years)	24.3 (2.0)	25.8 (3.1)	23.5 (2.6)	0.60
Monthly seizure frequency	7.5 (0.9)	4.9 (0.9)	8.5 (1.1)	**0.04**
QOLIE-31	35.2 (2.7)	38.2 (3.8)	33.5 (3.7)	0.43
				
*Intraoperative parameters*				
Mean arterial pressure	67.5 (1.7)	64 (3.0)	68.8 (2.1)	0.30
Heart rate	73.7 (2.2)	70 (4.1)	75 (2.7)	0.30
Respiratory frequency	11.6 (0.3)	11.7 (0.5)	11.5 (0.4)	0.88
				
*Biochemical analysis of the blood*				
pH	7.41 (0.07)	7.41 (0.01)	7.42 (0.01)	0.66
PCO_2_	28 (0.8)	29.6 (1.5)	28.1 (0.9)	0.35
HCO_3_	20.0 (0.3)	20.7 (0.6)	19.6 (0.3)	0.20
PO_2_	229 (11.0)	214.6 (25.1)	237.8 (10.3)	0.32
O_2_ saturation	99.7 (0.04)	99.6 (0.08)	99.7 (0.05)	0.90
Hematocrit	35.0 (0.7)	33.9 (1.4)	35.6 (0.8)	0.27
Hemoglobin	12.5 (0.9)	14.9 (2.9)	11.7 (0.3)	0.26
Glucose	116.3 (4.9)	103.4 (6.4)	121 (6.0)	**0.10**
Sodium	138.2 (3.9)	136.9 (1.2)	139 (1.7)	0.20
Potassium	4.1 (0.1)	4.2 (0.2)	4.1 (0.1)	0.63
Ionic calcium	4.2 (0.1)	4.1 (0.1)	4.2 (0.2)	0.95
Magnesium	0.4 (0.08)	0.4 (0.02)	0.5 (0.01)	0.15
Lactic acid	2.1 (0.2)	1.7 (0.3)	2.3 (0.3)	0.22
Storage time of samples (months)^a^	24 (1.5)	28.2 (2.9)	21.5 (1.7)	**0.05**
Time since last seizure (hours)^d^	225 (82)	200 (63)	239 (122)	0.82
Time for CX sampling (min)^e^	188.3 (7.1)	184.8 (10.3)	190 (9.7)	0.71
Time for AMY/HIP sampling (min)^f^	260.1 (10.0)	254.6 (16.9)	262.8 (12.8)	0.78
Time of HIP manipulation (min)^g^	11.2 (0.9)	11.7 (1.3)	11.1 (1.2)	0.70

Abbreviations: AMY, amygdala; CX, middle temporal neocortex gyrus; HIP, hippocampus; MRI, magnetic resonance imaging; QOLIE-31, Quality of Life in Epilepsy Inventory-31.

a Time course since brain tissue sampling and the homogenization for neurochemical evaluation (range 8 to 39 months).

b Anxiety disorders: generalized anxiety disorder (two patients in the non-dexamethasone group), social phobia (one patient in non-DEX group).

c Other psychiatric conditions: three patients with dysphoric disorder of epilepsy (one in the non-DEXA group), two patients with postictal psychosis (in the non-DEXA group), one patient with postictal anxiety (in the DEXA group).

d Time course since the last seizure attack occurrence and brain tissue sampling (range 12 to 590 h).

e Time course since anesthesia induction until CX tissue sampling (range 119 to 255 min).

f Time course since anesthesia induction until AMY/HIP tissue sampling (range 160 to 360 min).

g Time course since HIP vessels thermo-coagulation started until the complete resection of the HIP (range 5 to 25 min). Variables showing a '*P*' level of significance <0.15 are in bold.

**Table 2:** Comparison of phospho-GluA1-Ser831 and phospho-GluA1-Ser845 levels variation in the middle temporal neocortex, amygdala and head of the hippocampus according to dexamethasone treatment

*Variables*	*All cases*, n=*31, mean (s.e.)*	*No dexamethasone*, n=*11, mean (s.e.)*	*Dexamethasone*, n=*20, Mean (s.e.)*	P*-value*
*Middle temporal neocortex*				
P-GluA1-Ser831	118.6 (3.2)	126.9 (6.3)	114.3 (3.2)	0.06^a^
P-GluA1-Ser845	112.4 (3.0)	103.1 (4.1)	117.3 (3.6)	0.02^b^

*Amygdala*				
P-GluA1-Ser831	109.3 (3.0)	122.8 (4.6)	101.9 (2.8)	0.0003^c^
P-GluA1-Ser845	108.1 (3.3)	108.2 (6.0)	108.0 (4.1)	0.98

*Hippocampus*				
P-GluA1-Ser831	97.0 (3.7)	105.2 (7.2)	92.5 (4.0)	0.10^d^
P-GluA1-Ser845	104.2 (3.6)	100.5 (7.8)	106.3 (3.7)	0.46

a Non-significant trend of 12.6% decrease in the neocortex levels of P-GluA1-Ser831;

b Non-significant trend of 14.2% decrease in the neocortex levels of P-GluA1-Ser845;

c Significant decrease of 20.9% in the amygdala levels of P-GluA1-Ser831;

d Non-significant trend of 12.7% decrease in the hippocampus levels of P-GluA1-Ser831.

**Table 3:** Variables associated with amygdala levels of phospho-GluA1-Ser831

*Variables*	*Amygdala levels of phospho-GluA1-Ser831*			P-*value*
	*Univariate analysis*^a^			
*Gender*	*Mean (s.e.)*			
Female	103 (3.6)			
Male	117.4 (4.5)			0.02^a^
				
*AEDs regimen*				
Monotherapy	114 (5.0)			
Two AEDs or more	109 (3.9)			0.51^a^
				
*Phenobarbital treatment*				
No	111.7 (3.8)			
Yes	105.4 (4.9)			0.32^a^
				
	B	r	r^2^	P-value
*Univariate linear regressions*				
Monthly seizure frequency	−0.71	0.21	0.05	0.26^b^
Serum glucose	−0.19	0.32	0.10	0.12^b^
Storage time of samples (months)	0.36	0.19	0.04	0.30^b^
Dexamethasone dose (mg kg^−1^)	−145.6	0.69	0.48	0.00002^b^
*Multiple linear regression*				
Constant	134.8			<0.00001
Female	−0.90			0.85
Serum glucose	−0.10			0.33
Dexamethasone dose (mg kg^−1^)	−131.1			0.0003
		0.73	0.53	0.001^c^

Abbreviation: AEDs, antiepileptic drugs.

a Univariate analysis by Student's *t-*test;

b Univariate analysis by linear regression;

c Level of significance for the multiple linear regression model.

**Table 4:** Effects of dexamethasone treatment on signal transduction molecules, glutamate receptors and transporters, and astrocyte markers in the amygdala according to dexamethasone treatment

*Signal transduction molecules, astrocyte markers, and glutamate receptors and transporters*	*No dexamethasone,* n=*11, mean (s.e.)*	*Dexamethasone,* n=*20, mean (s.e.)*	P*-value*^a^
*Enzymes acting directly upon GluA1-Ser831 site*			
PP1ca	94.6 (2.8)	94.8 (2.0)	0.82
PKC activity	122.6 (5.5)	119.9 (5.4)	0.76
P-CaMKII	127.6 (9.9)	105.2 (3.7)	**0.02**^b^
Total CaMKII	93.4 (2.4)	100.2 (2.4)	0.08^a^

*Enzymes not acting upon GluA1-Ser831 site*			
PKA activity	120.9 (5.8)	125.1 (6.5)	0.67
P-ERK1	98.6 (5.1)	91.6 (3.9)	0.29
P-ERK2	96.6 (2.4)	98.8 (3.4)	0.87
P-JNK1	99.8 (3.6)	100.4 (4.2)	0.93
P-JNK2	100.68 (4.4)	99.5 (95.5)	0.89
P-AKT	95.6 (2.4)	95.5 (2.0)	0.75
P-p38	102.8 (2.8)	105.3 (2.3)	0.52

*GLU receptors sub unities and transporters, and astrocyte markers*^c^			
GluA1	96.8 (3.8)	97.8 (2.8)	0.83
GFAP	110.7 (4.0)	106.8 (1.9)	0.35
EAAT1	90.2 (4.3)	95.5 (3.5)	0.36
EAAT2	98.1 (6.4)	94.1 (5.4)	0.65

Abbreviations: AMY, amygdala; DEXA, dexamethasone

a There was a trend (*P*=0.08) for a presence of higher levels of total CaMKII in the AMY of DEXA in comparison to non-DEXA patients;

b Concerning the enzymes affecting the P-GluA1-Ser831 levels, DEXA treatment decrease 22.4% of P-CaMKII levels in the AMY, without affecting the PKC activity, P-CaMKII or PP1ca levels;

c GluA1=Levels of GluA1 subunit of glutamate AMPA receptor; GFAP=Levels of Glial Fibrilary Acidic Protein; EAAT1 and EAAT2=Levels of glial excitatory amino acid transporter (EAAT) type 1 and 2.
