# Supplementary material for: A single high dose of dexamethasone affects the phosphorylation state of glutamate AMPA receptors in the human limbic system
Source: Transl Psychiatry. 2016 Dec 13;6(12):e986–. doi: 10.1038/tp.2016.251 (PMC5290343; doi:10.1038/tp.2016.251)
Supplement: Supplementary Information [file tp2016251x1.doc]

**Figure 1**


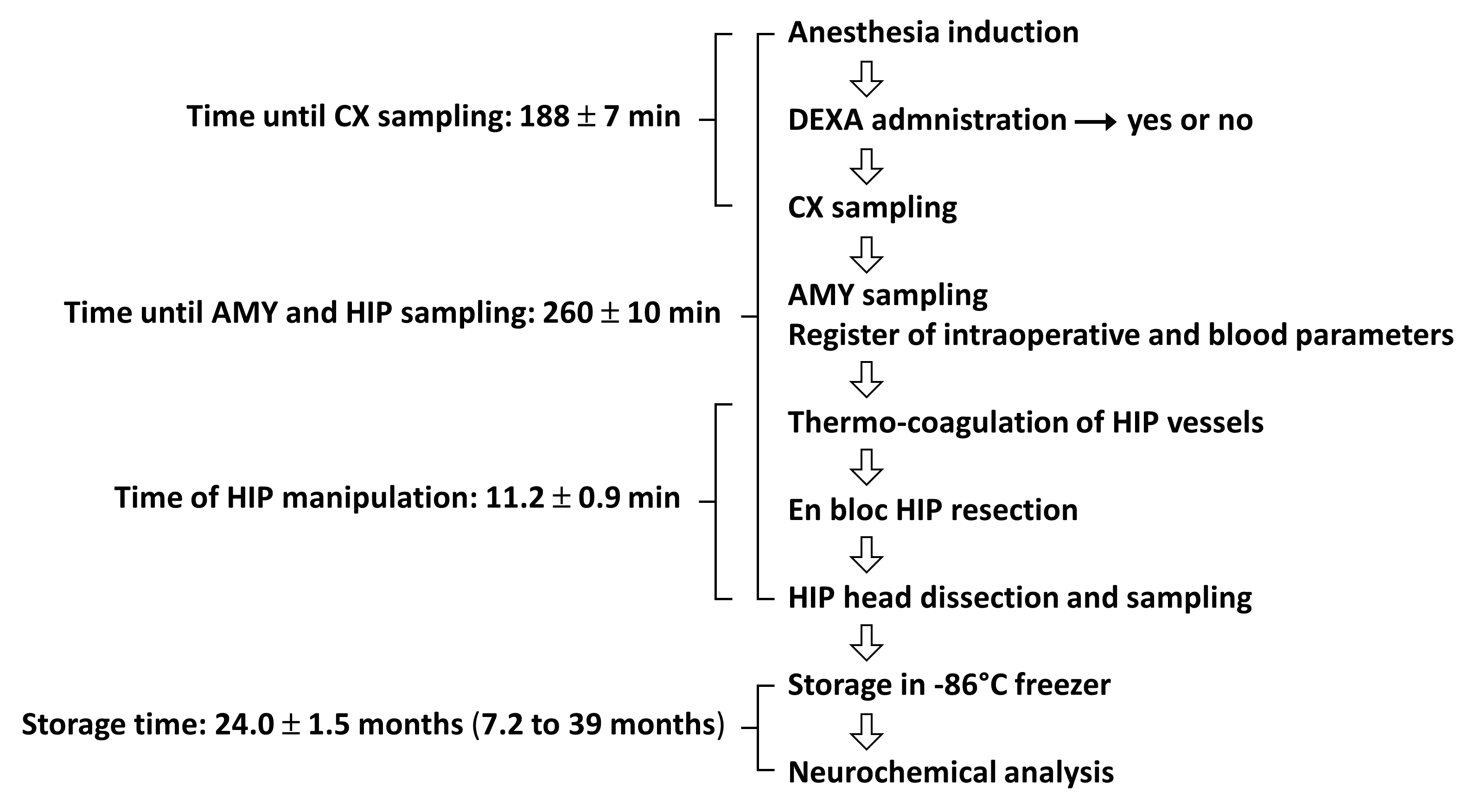


**Figure 1:** The sampling course of brain tissue collection. DEXA = dexamethasone. CX = middle temporal neocortex. AMY = amygdala. HIP = hippocampus.

**Figure 2A**


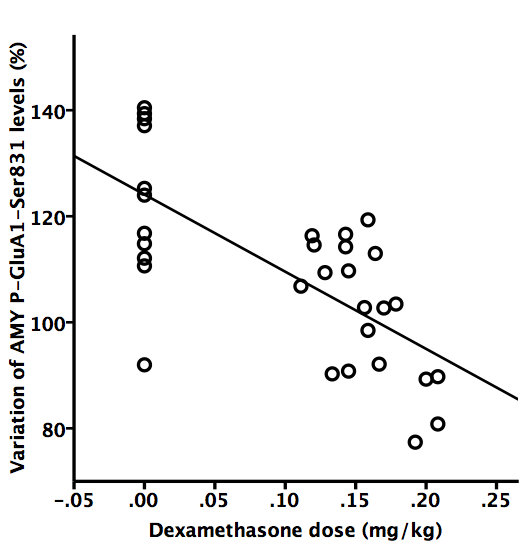


**Figure 2B**


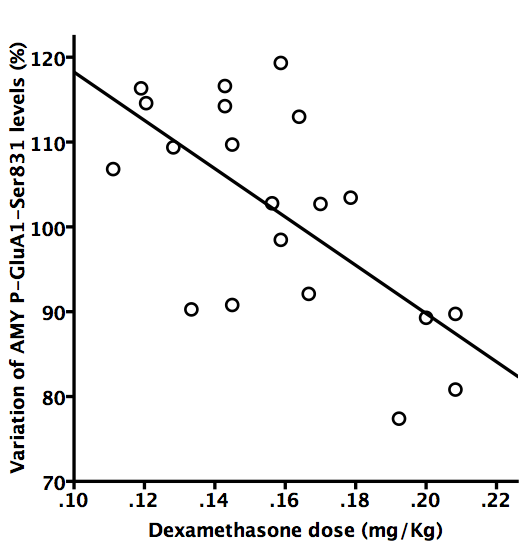


**Figure 2:** **A**: Linear regression showing a dose dependent effect of dexamethasone on phospho-GluA1-Ser831 levels in the amygdala (AMY), all patients (*n* = 31, r = 0.69; r^2^ = 0.48; p = 0.00002). **B**: The same analysis including only patients who received dexamethasone (n = 20, r = 0.66; r^2^ = 0.43; p = 0.002).

**Figure 3**


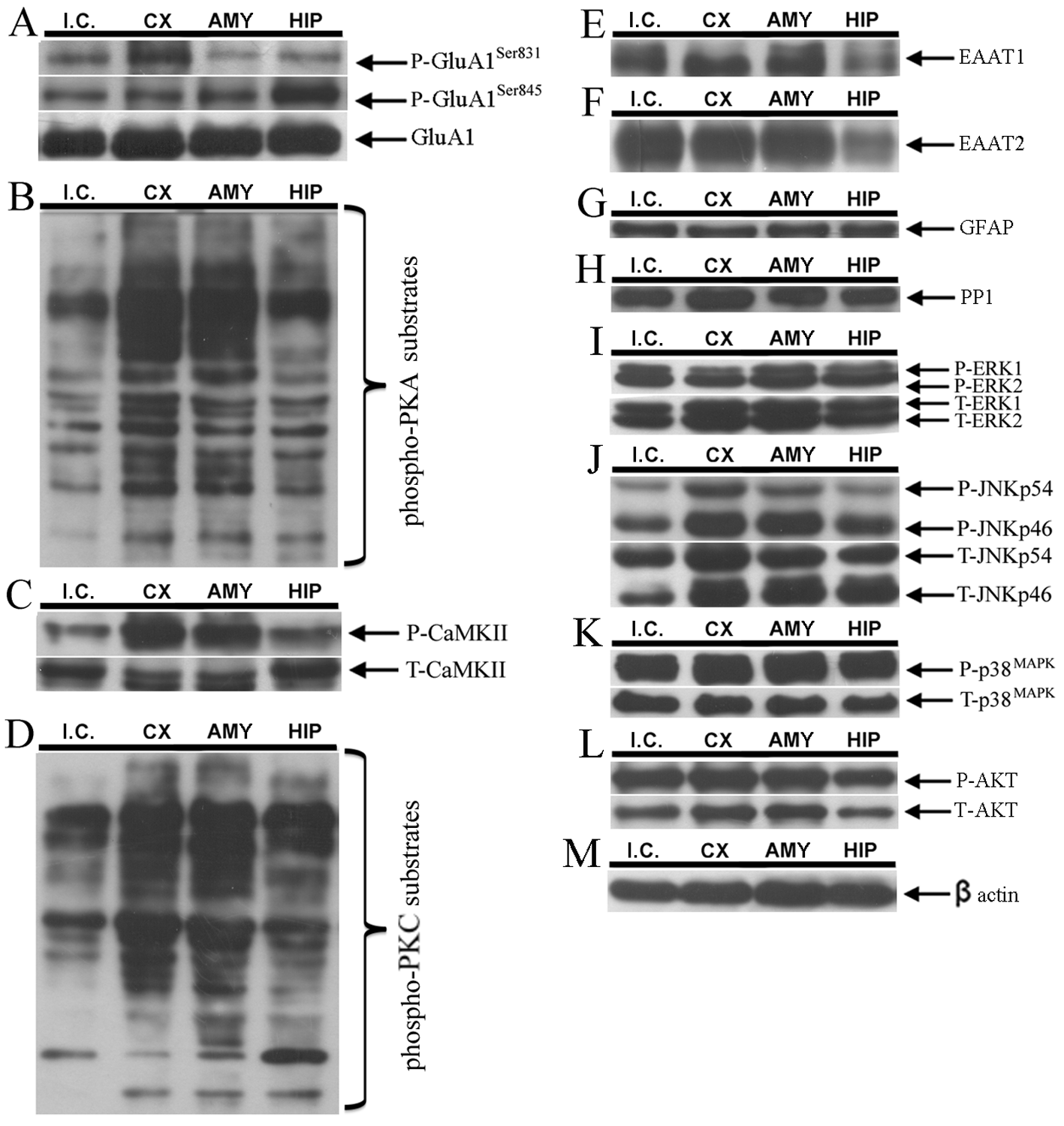


**Figure 3:** Representative western blots of GluA1 subunit of AMPA receptor (A), PKA (B), CaMKII (C), PKC (D), EAAT1 (E), EAAT2 (F), GFAP (G), PP1 catalytic subunit (H), ERK (I), JNK (J), p38^MAPK^ (K), AKT (L), β actin (M) in the middle temporal neocortex (CX), amygdala (AMY) and head of hippocampus (HIP) of patients and the internal control sample (IC). The images are illustrative and represent the pattern detection of targets of interest.
